# Localization of the Catalytic Domain of Copepod Luciferases: Analysis of Truncated Mutants of the *Metridia longa* Luciferase

**DOI:** 10.3390/life13051222

**Published:** 2023-05-21

**Authors:** Svetlana V. Markova, Marina D. Larionova, Igor A. Korotov, Eugene S. Vysotski

**Affiliations:** 1 Photobiology Laboratory, Institute of Biophysics SB RAS, Federal Research Center “Krasnoyarsk Science Center SB RAS”, Krasnoyarsk 660036, Russia; smarkova@mail.ru (S.V.M.); larionova.marina@inbox.ru (M.D.L.); igorkorotov96@gmail.com (I.A.K.); 2 School of Fundamental Biology and Biotechnology, Siberian Federal University, Krasnoyarsk 660041, Russia

**Keywords:** bioluminescence, coelenterazine, copepod luciferase, *Metridia* luciferase, catalytic domain, mammalian expression

## Abstract

Luciferases from copepods *Metridia longa* and *Gaussia princeps* are successfully used as bioluminescent reporters for in vivo and in vitro assays. Here, we report the minimal sequence of copepod luciferases required for bioluminescence activity that was revealed by gradual deletions of sequence encoding the smallest MLuc7 isoform of *M. longa* luciferase. The single catalytic domain is shown to reside within the G32-A149 MLuc7 sequence and to be formed by both non-identical repeats, including 10 conserved Cys residues. Because this part of MLuc7 displays high homology with those of other copepod luciferases, our suggestion is that the determined boundaries of the catalytic domain are the same for all known copepod luciferases. The involvement of the flexible C-terminus in the retention of the bioluminescent reaction product in the substrate-binding cavity was confirmed by structural modeling and kinetics study. We also demonstrate that the ML7-N10 mutant (15.4 kDa) with deletion of ten amino acid residues at the N-terminus can be successfully used as a miniature bioluminescent reporter in living cells. Application of a shortened reporter may surely reduce the metabolic load on the host cells and decrease steric and functional interference at its use as a part of hybrid proteins.

## 1. Introduction

**Figure 1:**
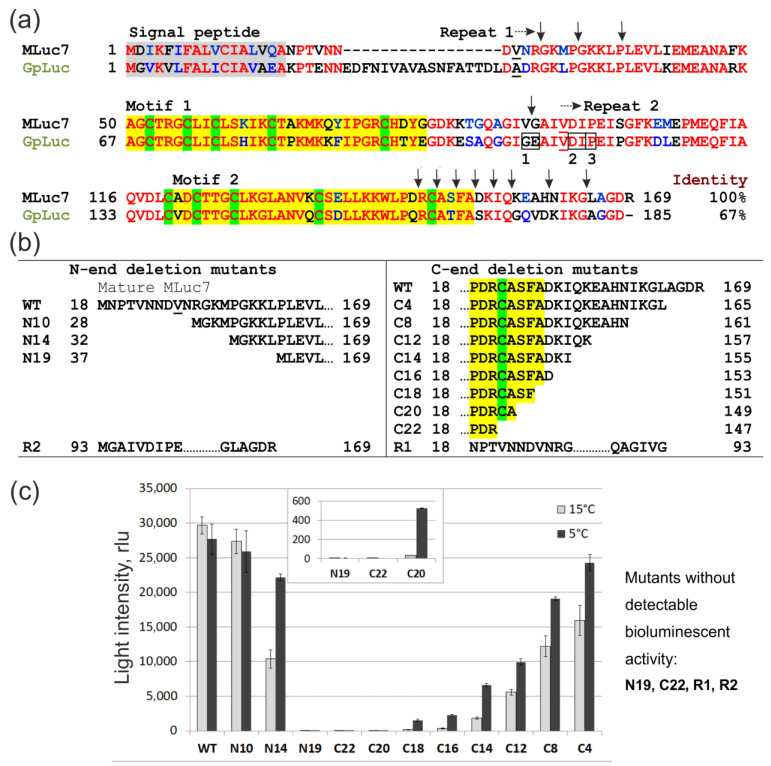
Structure and bioluminescent activity of MLuc7 deletion mutants. (**a**) Sequence alignment of MLuc7 and GpLuc (GenBank accession numbers: AJC98141, AAG54095, respectively). The identical amino acid residues are marked in red, and the ones with similar properties are marked in blue. The most similar motifs within non-identical repeats are marked in yellow, and conservative Cys residues are highlighted in green. Vertical arrows mark the boundaries of the MLuc7 deletion mutants. The sites dividing GpLuc into specific fragments are framed: 1—for protein complementation assay between Gly109 and Glu110 [15], 2—fragments with declared bioluminescent activity [9], 3—the end of the first half of truncated GpLuc with declared high activity [14]; (**b**) Sequences and designations of the MLuc7 deletion mutants. Numbers in mutant names indicate the number of deleted residues and terminus of mature protein from which these residues were deleted; (**c**) Activity of the MLuc7 mutants in crude lysates of *E. coli* cells measured at 15 °C (light gray) and at 5 °C (dark gray). The activity is normalized to the culture optical density. Inset shows an enlarged fragment of the graph for low-activity mutants. rlu: relative light units.

The secreted luciferase from marine copepod *Metridia longa* (MLuc) catalyzes oxidative decarboxylation of the substrate, coelenterazine, without involving any cofactors, which results in light emission with λ_max_~485–488 nm [1]. On account of high activity, simplicity of bioluminescent reaction, and stability, MLuc was successfully applied as in vivo and in vitro reporter shortly after being cloned [1,2]. Currently, application of MLuc continues to expand, and this is the same as of its homologous luciferase, GpLuc (Figure 1a), from the copepod *Gaussia princeps* [3]. Despite the growing use of these luciferases as reporters in biomedical research [2,4], many questions regarding their catalytic structure and luminescent properties remain unanswered, sometimes due to inconsistencies in the published data. The main hindrance here is the production of natively folded disulfide-rich luciferases by employing an inexpensive bacterial expression system where a reductive environment impedes the formation of proper S-S bonds.

To date, at least four groups of isoforms for MLuc derived from different groups of paralogous genes differing in size (16.5–22.2 kDa for mature proteins), sequence identity (54–81%), and some other features have been identified [1,5,6,7]. The multiple sequence alignment of MLuc paralogs, GpLuc, and other copepod luciferases clearly reveals a highly variable N-terminus, constituting up to one third of the amino acid sequence length of the longest luciferase and the C-terminal highly conservative region (Figures S1 and S2).

The variable N-terminus is not essential for bioluminescence, as is evident from the increased bioluminescent activity observed with the gradual removal of residues down to 15.1 kDa in the longest MLuc164 isoform (M5 in Figure S1) [8]. Thus, the bioluminescence catalytic function resides within the boundaries of the conserved C-terminal region. In copepod luciferases, this region is mainly formed by two non-identical tandem repeats, each of about 70 amino acid residues (Figure 1a) [1,9,10]. The repeat includes a highly conserved motif of thirty-two amino acids with five conserved cysteines, suggesting the presence of up to five disulfide bonds. An odd number of Cys residues within each repeat admits the existence of at least one disulfide bond between the repeats.

The presence of five disulfide bonds was found for isoform MLuc7 [6] and for GpLuc [11], but only four were revealed in the extremely psychrophilic isoform MLuc2 (Figure S1), with the optimum temperature for bioluminescence activity around 2–5 °C [7]. These intramolecular disulfide bonds are necessary for the functional activity of luciferases, because the addition of reducing agents quickly inactivates the enzyme [5]. Analysis of a series of point substitutions of cysteines in GpLuc also implies that the five correctly formed disulfide bonds are required for full bioluminescent activity [12].

The recently determined NMR spatial structure of GpLuc obtained in *Escherichia coli* cells has allowed disulfide bonds to be localized [13]. Two of them were found within the repeats, whereas the other three ones were formed by cysteines situated in different repeats. It is noteworthy that while the existence of one of them was derived from NMR data, another two S-S bonds were calculated from atomic distances and probability to form the bonds. Moreover, an attempt was made to localize the active site of GpLuc by using flexible docking of the coelenterazine molecule. According to modeling, the hydrophobic cavity was found where coelenterazine might be accommodated. It was also suggested that a variable N-terminus assists in the formation of the GpLuc substrate-binding cavity. These findings call into question the results on the bioluminescent activity of repeats of GpLuc (Figure 1a) separately expressed in *E. coli* cells [9,14] as the breaking of three disulfide bonds has to completely inactivate the enzyme, and only one hydrophobic cavity that would be suitable to serve as an active site is found in the protein structure. In addition, no noticeable bioluminescent activity in the same separate fragments of GpLuc corresponding to individual repeats was observed while developing the GpLuc-based complementation assay [15,16]. Furthermore, the supposition that the N-terminus of copepod luciferases might be involved in the formation of the substrate-binding cavity disagrees with the results obtained for homologous MLuc164 mutants with this part deleted [8]. The currently available data, as may be seen, are rather contradictory; consequently, it is hard to draw conclusions on the localization of the active site of copepod luciferases and the involvement of specific amino acid residues in the catalytic oxidation of coelenterazine.

Here, we report the identification of the minimal sequence required for the oxidative decarboxylation of coelenterazine and bioluminescent activity of copepod luciferase by studying a set of N- and C-terminal gradual-deletion mutants of the smallest copepod luciferase, MLuc7 isoform from *M. longa* [8]. The analysis of 3D-structural models of MLuc7 and its deletion mutants allowed us to suggest that α-helices 1 and 2 of copepod luciferases located within the N-terminal variable region are not essential for bioluminescence and do not participate in substrate catalysis. We also demonstrate that the ML7-N10 mutant with deletion of ten amino acid residues at the N-terminus can be successfully used as a miniature bioluminescent reporter in living cells.

## 2. Materials and Methods

### 2.1. Materials

Coelenterazine was obtained from NanoLight Technology, a division of Prolume Ltd. (Pinetop, AZ, USA). A stock coelenterazine solution was prepared in methanol and stored at −20 °C for several weeks. The GenBank accession numbers for the used MLuc7 and GpLuc luciferase sequences were AJC98141 and AAG54095, respectively.

### 2.2. Genetic Constructs

For direct expression of the luciferases and deletion mutants without signal peptides in *E. coli* cells, the pET22b+ plasmid (Novagen, Madison, WI, USA) was used; all sequences were provided with the initiation methionine. The plasmids for the MLuc7 deletion mutants (Figure 1b) were constructed by the following mechanisms: (i) amplification by specific primers (Table S1) with the desired changes and cloning sites with the following cloning in appropriate expression vector; and (ii) oligonucleotide-directed mutagenesis using the QuikChange site-directed mutagenesis kit (Agilent, Santa Clara, CA, USA). The pET22b+ plasmid (Novagen) was used for direct expression of the deletion mutants without signal peptides in *E. coli* cells; the sequences of these mutants were provided with the initiation methionine. The pET22b+-MLuc7 plasmid [6] served as a template DNA for bacterial expression variants. The pcDNA3m-MLuc7 plasmid [6] as a template DNA for mutagenesis and the pcDNA3m vector modified from pcDNA3.1+ (Invitrogen, Waltham, MA, USA) were used to create constructs that would provide the secreted expression of the luciferase mutants in mammalian cells through MLuc7 native signal peptides. The coding part of GpLuc without signal peptides was synthesized by PCR using pUC18-GLuc (NanoLight Technology, Pinetop, AZ, USA) as a template and cloned in NdeI-XhoI sites of pET22b+. The created constructs are shown in Figure 1b; the primer sequences are listed in Table S1. All constructs were confirmed by DNA sequencing performed in the SB RAS Genomics Core Facility (ICBFM, Novosibirsk, Russia).

### 2.3. Expression in E. coli, Purification, and Preparation of Natively Folded Luciferases

Preparations of high-purity natively folded mutant luciferases were obtained from *E. coli* inclusion bodies by oxidative refolding and chromatography purification. The *E. coli* cells BL21 CodonPlus (DE3)-RIPL (Stratagene, San Diego, CA, USA), harboring constructs with MLuc7 deletion mutants, were cultivated at 37 °C in LB medium supplemented with 200 µg/mL ampicillin. The luciferase synthesis was induced at OD_600_ = 1.2 with 1 mM IPTG, after induction, the cultivation was continued for 1 h. Cell harvesting, washing of inclusion body pellets, solubilization of luciferases, oxidative refolding, and chromatography purification were performed as described for MLuc7 [17] with certain modifications [18].

### 2.4. Expression of MLuc7 and Its Mutants in Mammalian Cells

The pcDNA3.1(+) (Invitrogen) plasmid was used to express MLuc7 and its mutants in CHO cells that were grown in 96-well plates in DMEM/F-12 medium (Gibco, Waltham, MA, USA) supplemented with 10% FCS (Gibco) at 37 °C and 5% CO_2_. The transient transfection of CHO cells (7000 cells/well) with a plasmid was conducted with Lipofectamine 2000 (Invitrogen) according to the manufacturer’s protocol. After 5 h, the transfection medium was replaced by fresh medium and from that moment on, luciferase secretion was monitored.

### 2.5. Bioluminescence Assay

Bioluminescence was measured using a temperature-stabilized single-cell luminometer BLM-003 (Oberon-K, Krasnoyarsk, Russia) by rapid injection of 5 µL of 0.2 mM coelenterazine solution in methanol into a cell containing a protein sample in 0.5 mL of the assay ML buffer (0.3 M NaCl, 0.015% gelatin, 50 mM Tris-HCl, pH 7.5). The luminometer was supplied with neutral density filters to extend the detection range. To measure the activity of the MLuc7 mutants in crude lysates of *E. coli* cells, the pellet from 2 mL of induced culture was disrupted by sonication in 0.2 mL of ML buffer and centrifuged. To determine thermostability, the MLuc7 samples were incubated in a block heater at indicated temperatures and then cooled on ice for 5 min before measuring the bioluminescence.

The assay of luciferase activity in response to the secondary addition of the substrate was performed in ML buffer supplemented with 0.02% NP-40 detergent for protein stabilization. The reaction kinetics were measured based on the protocol described in work [19]. The injection of 0.2 mM coelenterazine solution was repeated in 900 s after the initiation of bioluminescence.

The level of the luciferase expression in mammalian cells was estimated by the bioluminescent activity of picked medium aliquots (1–5 µL). Measurements were carried out with samples diluted in 100 µL of ML buffer and loaded into a 96-well plate using a Mithras LB 940 Multimode microplate Reader (Berthold Technologies) equipped with an auto-injector. The bioluminescence was recorded immediately after the injection of 50 μL freshly prepared coelenterazine diluted in ML buffer at a final concentration of 1 µM.

### 2.6. Spectral Measurements

Bioluminescence spectra were measured with a Cary Eclipse fluorescence spectrometer (Agilent Technologies) in the ML buffer and corrected for spectral sensitivity of the instrument. Bioluminescence was initiated by injection of 10 µM coelenterazine (protein/coelenterazine molar ratio was 1:100).

### 2.7. Structural Analysis

The predicted spatial structures of *M. longa* luciferase MLuc7, MLuc164, and theirs truncated variants N10 and M5, respectively, were obtained using the online server I-TASSER [20,21,22]. As a reference structure for prediction, the NMR structure of *G. princeps* luciferase, GpLuc, was chosen (PDB ID 7D2O) [13]. Further visualization and superposition of the protein structure, as well as amino acid number adjustment, were performed using Coot [23] and PyMOL 2.5.0 (Schrödinger, New York, NY, USA). The RMSD was calculated by the Align method of PyMOL 2.5.0; hydrophobicity coloring was made using a publicly available PyMOL script [24], based on the amino acid consensus hydrophobicity scale [25].

## 3. Results and Discussion

### 3.1. Functional Analysis of Deletion Mutants in Crude E. coli Lysates

One of the simplest ways to localize the enzyme catalytic domain(s) is to evaluate the catalytic ability of deletion mutants. MLuc7 is the smallest natural luciferase comprising the minimal variable N-terminus. In fact, this isoform consists almost entirely of two repeats which form the C-conserved region of copepod luciferases [2]. It makes MLuc7 a convenient model to localize the catalytic domain of copepod luciferases. The obtained set of N- and C-terminal gradual-deletion mutants for MLuc7 is schematically presented in Figure 1a,b with R1 and R2 corresponding to individual homologous repeats in the sequence. The deletion variants were directly expressed without signal peptides in *E. coli* cells by employing the pET expression system and were functionally compared using crude cell extract.

When synthesized in this expression system, MLuc7 of the wild type is mainly found in an insoluble fraction as inclusion bodies [15]. To avoid inaccuracies in the bioluminescent activity determination in view of possible changes in the levels of expression and solubility, those were analyzed for all mutants by gel electrophoresis (Figure S3). After induction of synthesis, all deletion mutants were found in the insoluble fraction of the *E. coli* lysate, similar to wild-type MLuc7, except for ML7-R2. In this case, half of the synthesized protein was in a soluble form. However, this was not significant because we could not detect any bioluminescent activity in separately expressed luciferase halves corresponding to the repeats (ML7-R1 and ML7-R2, Figure 1c). These findings are consistent with the absence of noticeable bioluminescent activity in the homologous halves of GpLuc in studies on the construction of its fragments for protein complementation assays [15,16], but rather differ from the data for GpLuc, where bioluminescent activity was declared for analogous fragments (Figure 1a) [9,13].

The bioluminescent activity of the mutants was evaluated at 15 °C (optimal for MLuc7 [6]) and 5 °C because the deletions may reduce the structural rigidity of the protein molecule and potentially decrease the reaction temperature optimum, which occurs in the case of the psychrophilic MLuc2 isoform from *M. longa* containing an additional glycine cluster in the amino acid sequence and four disulfide bonds only [7]. The N-terminal deletion of 10 residues (ML7-N10) did not significantly change the bioluminescent activity (Figure 1c), while removing 14 amino acids (ML7-N14) resulted in a loss of ~70% of activity at 15 °C and decrease in the temperature optimum of the reaction (mutant activity at 5 °C was ~20% less than that of wild-type MLuc7 at this temperature). The deletion of 19 amino acids (ML7-N19) completely destroyed the bioluminescent activity, thereby allocating the catalytic domain boundary between G32 and L37 from the N-terminus of MLuc7.

The deletions at C-terminus caused a gradual decrease in bioluminescent activity as well, which, to our surprise, partially remained even when the deletions were extended to the part of highly conserved motif 2 (Figure 1). A loss in bioluminescent activity for C-terminus deletion mutants with an increasing number of truncated amino acid residues was also accompanied by a significant decrease in the reaction temperature optimum—a higher bioluminescent activity was observed at 5 °C for all mutants. The activity of the ML7-C20 variant with the 3-aa deletion of the conserved motif 2, for example, could only be detected at 5 °C (Figure 1c, insert). Enzymatic activity of the deletion mutants could be detected as long as the highly conserved 148Cys was not deleted. Then, a complete loss of bioluminescent activity and bordering the catalytic domain by this 148Cys residue from the C-terminus side followed. Thus, the catalytic domain of MLuc7 luciferase resides within the G32-A149 sequence and both repeats are involved in its formation. Considering a high degree of identity between the conserved C-regions of copepod luciferases, this statement seems to be true for all of them. Interestingly, similar bordering has been recently identified for the artificial luciferase ALuc30 designed on the base of consensus sequences of the cloned copepod luciferases [26].

### 3.2. Bioluminescent Properties of High-Activity Deletion Mutants

One of the attractive features of any reporter is a small size, which provides the minimum of: (1) metabolic load on host-cell metabolism when it is used as a genetically encoded reporter; (2) steric hindrances when applying the one as a part of hybrid proteins; and (3) distance when the bioluminescent reporter is used as a BRET partner. These are the reasons we chose two deletion variants of MLuc7 with minimal effects from deletions on bioluminescent activity, ML7-N10, and ML7-N10C4 (Figure 1) for more detailed characterization. Monomeric preparations of the natively folded proteins were obtained from *E. coli* inclusion bodies by glutathione-based oxidative refolding, followed by chromatography purification (the purity exceeded 95%) (Figure S3c). The homogeneity of the preparations was confirmed by SDS and semi-native polyacrylamide gel electrophoresis.

**Figure 2:**
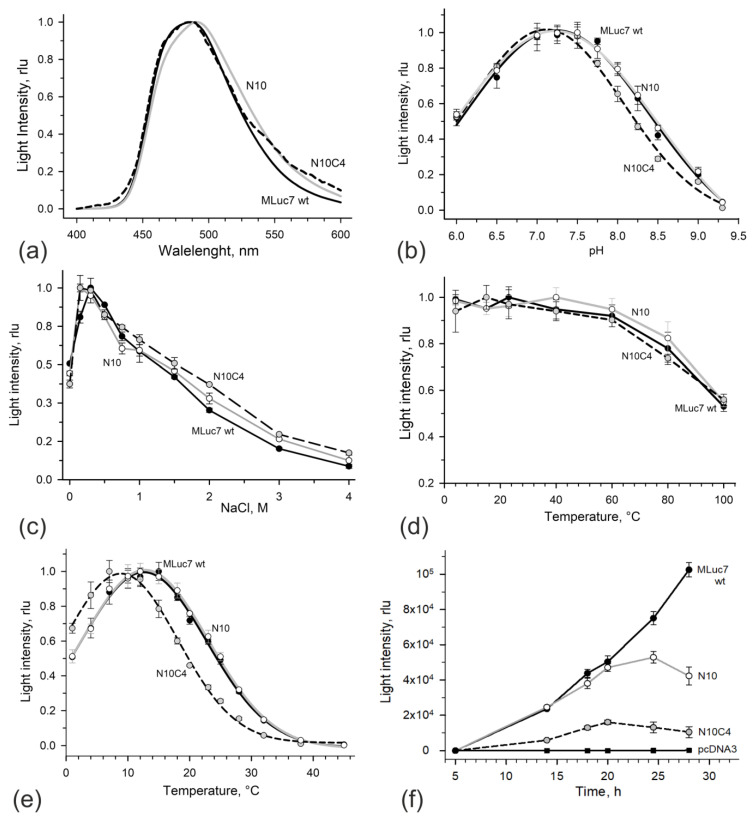
Bioluminescent properties of MLuc7 deletion mutants. (**a**) Bioluminescent spectra. (**b**) Effect of pH on bioluminescent activity. The buffer was 50 mM Bis-Tris propane of different pH with the addition of 0.3 M NaCl; (**c**) Effect of NaCl on bioluminescent activity. The buffer was 50 mM Tris-HCl pH 7.5. (**d**) Thermal stability of MLuc7 deletion mutants after 1 h incubation in ML buffer with 0.02% NP-40; (**e**) Effect of temperature on light intensity; (**f**) Time course of activity of MLuc7 deletion mutants secreted by CHO in culture media. The squares show the control light signals from culture medium of cells transfected with vector pcDNA3.1+ only. Black lines and circles correspond to wild-type MLuc7, gray lines and open circles correspond to ML7-N10 mutant, dashed lines and gray circles correspond to ML7-N10C4 mutant. Data are the mean ± SD. rlu: relative light units.

The bioluminescence spectrum of the ML7-N10C4 mutant with λ_max_ around 485 nm corresponded to those of wild-type MLuc7, the other *M. longa* isoforms, and GpLuc [1], while the one of the ML7-N10 mutant appeared to be a little wider and shifted by ~5 nm towards the longer wavelengths (Figure 2a). The similarity of light-emission spectra of MLuc truncated mutants and GpLuc indicates an identical environment of a substrate in their active centers and, consequently, it obviously shows that the variable N-terminus of both GpLuc and MLuc is hardly involved in the formation of the substrate-binding cavity of copepod luciferases as was suggested based on the NMR structure of GpLuc [13].

The pH profiles of light intensities of the luciferase mutant ML7-N10 and wild-type MLuc7 turned out to be identical at physiological pH in the range of 6.5–8.0, whereas the pH profile of the ML7-N10C4 mutant was slightly shifted towards acidic pH by ~0.25 units (Figure 2b). The dependences of bioluminescent activities on salt concentration of both mutants are also almost identical to that of MLuc7 refolded from *E. coli* inclusion bodies, i.e., the optima were found at ~0.3 M (Figure 2c). However, of note is that ML7-N10C4 appeared to be somewhat more resistant to high salt concentrations. The distinctive feature of all known copepod luciferases is a greater resistance to thermal inactivation due to the presence of multiple disulfide bonds [2]. Both deletion mutants have retained this property (Figure 2d), thus indicating the presence of the correctly formed S-S bonds in both mutants.

Bioluminescence activity is a parameter that is more affected by simultaneous truncation of residues at the N- and C-termini. The ML7-N10C4 mutant retains only 23% of activity compared to wild-type MLuc7 (Table 1). In contrast, the deletion of 10 residues at the N-terminus only does not influence bioluminescent activity (ML7-N10 mutant preserves practically the same activity as the wild-type MLuc7). The low activity of the ML7-N10C4 mutant can be also caused by a non-optimal temperature of 15 °C because the deletion of four residues at the C-terminus results in a shift in the temperature optimum of bioluminescent reaction from 15 °C to 7–10 °C (Figure 2e).

In this way, the removal of the first ten residues in mature MLuc7 does not practically affect the bioluminescence properties of luciferase. However, additional deletion of even four amino acids at the C-terminus significantly reduces activity and shifts the temperature optimum. This emphasizes the importance of the C-terminus in stabilizing the molecular structure.

To test the truncated MLuc7 variants as secreted bioluminescent reporters, the deletion mutants ML7-N10 and ML7-N10C4 in the pcDNA3m vector were transiently transfected to CHO cells. The plasmids encoding the truncated versions of MLuc7, corresponding to the ML7-R1 and ML7-R2 repeats (Figure 1b) with N-terminal signal peptides, were also tested for transient transfection of mammalian cells. The cells were observed to start secreting the reporter immediately after transfection and to accumulate the luciferase in the medium. Figure 2f shows the time course of secretion after replacement of the transfection medium to the fresh medium after 5 h treatment. Up to the 20 h point, bioluminescent activity grew synchronously for all three luciferases. It should be noted that the intensity of light signals was in proportion to those determined for high-purity proteins (Table 1). Then, the increase in activity slowed down and began to fall for the mutant variants, while for wild-type MLuc7, it continued. Most likely, such a pattern for deletion mutants is associated with partial degradation in the medium due to its lower resistance to the action of proteases compared to the wild-type luciferase.

It is noteworthy that each of the MLuc7 repeats itself (ML7-R1 and ML7-R2) did not reveal any bioluminescent activity exceeding that of the negative control, namely the pcDNA3.1+ vector without insert. This is additional evidence for the results obtained on the bacterial expression of halves of MLuc7 luciferase and for the absence of bioluminescent activity in separate domains of luciferase corresponding to the ML7-R1 and ML7-R2 repeats, as was previously suggested [9,14].

### 3.3. Protein Spatial Structure Analysis

Despite active development of numerous copepod luciferase-based reporters, the structural features of luciferases themselves remained unknown until recently, and these were only derived from prediction structural analysis, spectral studies, or mutagenesis [14,27,28,29,30] that could indirectly determine the involvement of certain amino acids in the enzyme active center. Because copepod luciferases have no significant amino acid sequence homology with other proteins, thereby representing a unique class of proteins [2], the exact tertiary structure of luciferases could not be predicted for a long time.

**Figure 3:**
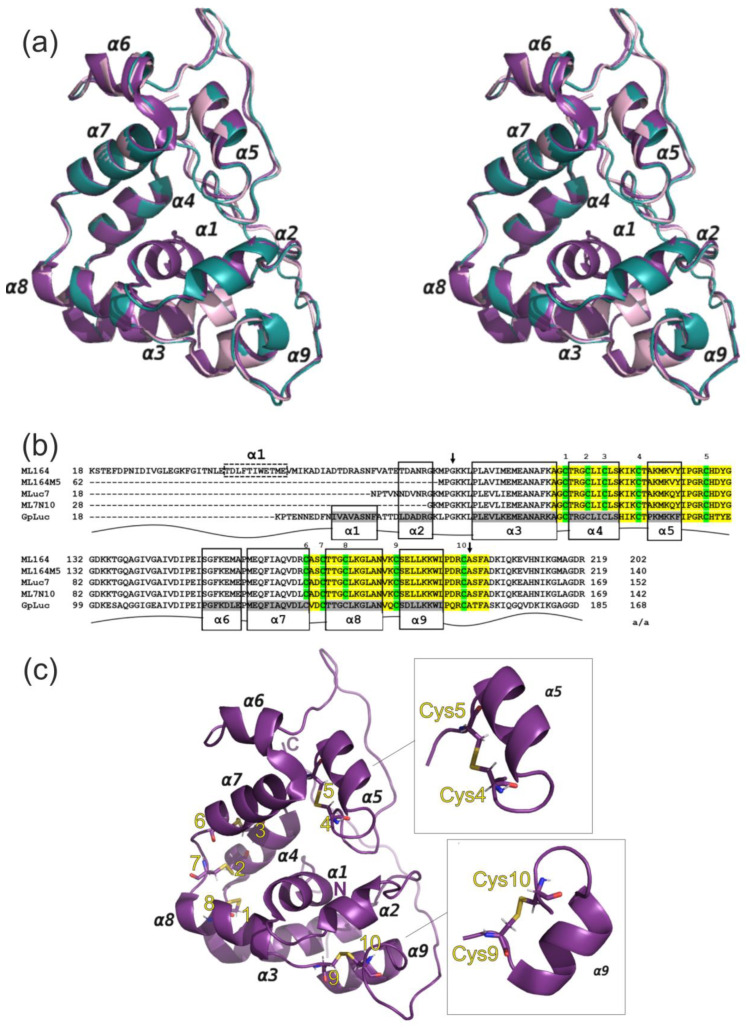
(**a**) Overall structure of GpLuc (PDB ID 7D2O) aligned with structures of MLuc7 and ML7-N10 mutant predicted by online server I-TASSER [22,23,24] presented as a stereo capture. GpLuc is shown in purple, predicted model for MLuc7 is shown in aquamarine, and predicted model for ML7-N10 is shown in pale pink. α-helixes are indicated by numbers 1–9 as it was ordered in the study [13]; (**b**) Amino acid alignment of GpLuc and isoforms of Metridia luciferase, MLuc164, MLuc7, and their truncated mutants, ML164M5 [8] and ML7-N10, respectively. The residues are numbered in accordance with full-length protein sequences. The conservative motifs are highlighted in yellow, and cysteines are colored in green and numbered. Gray boxes indicate the α-helixes according to the structure of GpLuc. Due to high homology within luciferases’ sequences, the similar patterns in frames were expanded to all aligned sequences. Because of low-confidence score for prediction of the full-length MLuc164 model, the N-terminal α1-helix (in dashed box) was identified using PSSpred server, providing the prediction of protein secondary structures [32]. The a/a column represents the length of amino acid sequences for mature proteins without signal peptides. The arrows show the boundaries of the sequence (32-149) found as a minimal essential region for catalytic activity of MLuc7; (**c**) Spatial structure of GpLuc with determined disulfide bridges. Insert shows loop-α5-helix-loop and loop-α9-helix-loop patterns forming the folds in the same manner.

However, in 2020, the NMR structure of copepod luciferase, GpLuc, was determined (PDB ID 7D2O) (Figure 3a) [13]. For NMR experiments GpLuc was obtained in a soluble form by bacterial expression in an M9 medium containing ^13^C and ^15^N and by the subsequent oxidative refolding on air [31]. This NMR structure is considered a novel coelenterazine-dependent luciferase fold which represents a globe-like protein formed by nine α-helices (α1–α9) and disordered regions at the N- and C-termini and at the region between two conservative motifs of luciferase (Figure 3a).

The structure of GpLuc allowed us to predict the spatial structure of Metridia luciferase. In the present study, this known structure was used as a reference model for predicting the structures of MLuc7 luciferase and its highly active truncated mutant, ML7-N10 (Table 1), using the online server I-TASSER [22,23,24] (Figure 3a). The predicted models for MLuc7 and ML7-N10 (Coordinates data S1, S2) showed high confidence with C-scores [22,23,24] of 1.28 and 1.86, respectively. The RMSD values were calculated for MLuc7 and ML7-N10 with alignment to GpLuc as 0.957 Å and 0.792 Å, respectively. The aforementioned parameters allow one to consider the obtained high-confidence models of Metridia luciferases as suitable structures for further analysis.

The overall structures of the obtained Metridia luciferase models in general corresponded to the fold of GpLuc because of high homology (Figure 3a and Figure S1). The Metridia structures, such as that in GpLuc, also have a set of α-helices and intrinsically disordered regions located at C-terminus, as well as between helices α5 and α6 (Figure 3a,b), which corresponds to the sequence between two conservative motifs (Figure 1a). The main difference in the structural organization of the two luciferases is the absence of the central α1-helix in the natural sequence of MLuc7 (Figure 3a,b). This fact clearly confirms that this structural element does not play an essential role for enzymatic activity in copepod luciferases.

Two of the obtained mutants, ML7-N10 and ML7-N14, with the deleted first 10 and 14 N-terminal amino acid residues (Figure 1b), represent truncated versions of MLuc7 without α2-helix. According to our results, these deletions caused no sufficient changes in bioluminescence (Figure 1c). However, the truncation in ML7-N19 that affected the first proline of the α3-helix (Figure 3b) resulted in complete inactivation of the enzyme. Considering the data on the M5 mutant of MLuc164, which had a deleted variable N-terminus and exhibited even a two-fold increase in bioluminescent activity (Figure 3b) [8], we suggest that the N-terminal boundary of the catalytic domain of copepod luciferases lies at the edge of the α3-helix. As such, the conclusion could be that helices α1 and α2 are not involved in the oxidative decarboxylation of coelenterazine.

The disulfide bonds in the structures of copepod luciferases are of key importance in bioluminescence, considering that the addition of DTT to the luciferase samples results in their inactivation [33]. Original structural analysis on GpLuc yielded the positions of disulfide bonds that connect conservative motifs containing 10 Cys residues. It suggests that the combination of disulfide bonds may be the same for the whole class of copepod luciferases. The NMR structure made it possible to clearly identify the pairs of cysteines forming the S-S bridges: 1/3, 4/5, and 9/10. However, the contacts 1/8 and 2/7 were calculated according to the atomic distances between Sγ atoms. Interestingly, the three of these pairs in the same form were predicted by DISULFIND [14]. The structure reveals that disulfide bridges (1/8, 3/6, and 2/7) firmly bind antiparallel bundles, thereby tightening the α3/α8 and α4/α7 helices (Figure 3c). However, the two well-defined loop-α-helix-loop patterns for α5 and α9 adopt similar folds, where the helices encompass the residues between the two cysteine pairs located at the end of each of the conserved motifs (Figure 1a and Figure 3b,c). The similarity in the fold of α5 and α9 helices may confirm the hypothesis on the evolution of copepod luciferases originated by the duplication of the gene encoding consensus sequences in motifs 1 and 2 [10]. The importance of the cysteine No. 5 in α5-helix was already confirmed by mutagenesis that resulted in complete inactivation of GpLuc [27]. The current study also determined that the truncation of the last cysteine placed at α9 in ML7-C22 mutant leads to the loss in bioluminescence activity. Numerous truncations of the C-terminus of Metridia luciferase definitely show the importance of the last cysteine, providing the stability of the protein molecule, and, consequently, the enzyme activity. Considering the above, we can conclude that the ability of copepod luciferase to catalyze the bioluminescence reaction remains as far as at least cysteines No. 4, 5, and 9, 10, and the structural elements loop-α5-helix-loop and the last loop-α9-helix-loop are present in the sequence. Thus, it was determined that the C-terminal boundary of the catalytic domain of copepod luciferase is placed at the edge of the last cysteine.

Based on the predicted structures, we also attempted to determine the reason for the ~5-fold reduction in luciferase activity observed in the truncated mutant ML7-N10C4, which differs from ML7-N10 by only four C-terminal residues. The C-terminus represents the most conserved region among all known copepod luciferases and terminates a second tandem repeat. It belongs to intrinsically disordered regions with high flexibility, as it was shown for GpLuc [13]. The decrease in activity observed in ML7-N10C4 leads us to suggest that the C-terminal unstructured region is functionally important. In a recent study investigating the mechanism of bioluminescence of another coelenterazine-utilizing luciferase, Renilla, the presence of a cap domain was discovered, providing an open or closed state for the substrate cavity [34]. It should not be excluded that the C-terminal part of the copepod luciferases may similarly serve as a structural element, providing a hydrophobic “wall” for the substrate-binding pocket.

**Figure 4:**
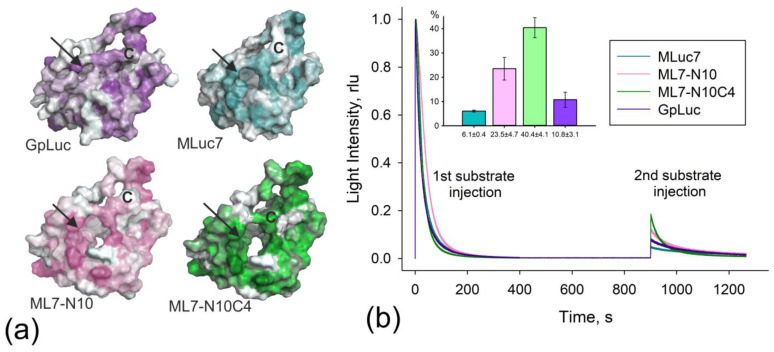
(**a**) The interfaces of copepod luciferases shown in a hydrophobic gradient: from non-hydrophobic gray to hydrophobic amino acids colored. The entrance into substrate-binding pocket is indicated by arrows; (**b**) The normalized bioluminescent reaction kinetics of MLuc7, ML7-N10, ML7-N10C4, and GpLuc in response to the first and second injections of 0.2 mM coelenterazine in methanol (5 µL) are shown. The injections were repeated after 900 s. The protein concentrations in the mixtures were approximately 10 nM. The insert displays the maximum light signal observed after the second portion of the substrate, given as a percentage. The measurements were conducted in triplicate. Data are the mean ± SD. rlu: relative light units.

Structural modeling of MLuc7, ML7-N10, and ML7-N10C4 as surfaces with indicated hydrophobic regions revealed significant differences in the hydrophobic pockets as compared to the previously published GpLuc structure. While original structural analysis of GpLuc postulated an entrance to the substrate cavity between α4, α5, and the C-terminus, in the case of ML7-N10 and ML7-N10C4, this cavity was extended throughout the entire molecule, forming a distinctly gaping hole (Figure 4a). We supposed that this may not only affect the bioluminescence activity of the deleted luciferase mutants but also their ability to retain the reaction product, coelenteramide, in the substrate-binding pocket.

This hypothesis was experimentally tested by evaluating the luciferase activity upon addition of the second portion of the substrate coelenterazine into the reaction mixture after bioluminescence was initiated. As was previously shown for GpLuc [19], the maximum bioluminescent activity at the second injection of the substrate was around 10%. In this study, we evaluated the activity after secondary substrate injection for MLuc7, ML7-N10, and ML7-N10C4 in comparison to GpLuc under the same conditions. The activity values measured after secondary substrate injection for MLuc7 and GpLuc were 6.1 ± 0.4% and 10.8 ± 3.1%, respectively, which is consistent with the previous data, and show that the hydrophobic substrate cavity is still occupied by coelenteramide. Surprisingly, for the deleted Metridia luciferase mutants, bioluminescence after a second injection of the substrate reached 23.5 ± 4.7% for ML7-N10 and 40.4 ± 4.1% for ML7-N10C4 (Figure 4b). These findings clearly indicate that the N-terminal amino acid residues, which form the α2-helix, and C-terminal intrinsically disordered region are functionally important for the formation of a cavity that retains the reaction product but are not involved in the catalytic domain of the copepod luciferases.

## 4. Conclusions

In summary, we report for the first time the minimal sequence of *M. longa* luciferase that ensures its bioluminescence activity. One was achieved by successive truncations of the sequence of the smallest copepod luciferase, the MLuc7 isoform. The catalytic domain was found within G32-A149 of highly conserved the MLuc7 sequence formed by both non-identical repeats. The 3D-structural modeling revealed that the N-terminal boundary of the catalytic domain of copepod luciferases lies at the edge of the α3-helix, and consequently, the helices α1 and α2 identified for Gaussian luciferase do not participate in the oxidative decarboxylation of coelenterazine. In contrast to the N-terminus, the C-terminal sequence of Metridia luciferase was found to play an important role in retaining the high level of bioluminescent activity. Taking into account the high homology of this part amongst the known copepod luciferases, we suggest that the boundaries defined for the catalytic domain of Metridia are the same for all copepod luciferases. In addition, we characterized the bioluminescent properties of two deletion mutants and showed that the 15.4 kDa ML7-N10 mutant can be successfully used as a small bioluminescent reporter that would yield a reduction in the metabolic load on the host cell in in vivo studies and a decrease in steric and functional interference at its use as a part of hybrid proteins.

## Figures and Tables

**Table 1:** Bioluminescence activities of MLuc7 and its mutants.

Protein	Initial Bioluminescence Activity ^1^ (rlu/mg, ×10^9^)	Relative Bioluminescence Activity (%)
MLuc7	12.9 ± 1.4	100
ML7-N10	11.8 ± 1.1	92
ML7-N10C4	3.0 ± 0.2	23

^1^ Bioluminescence was measured at 15 °C.

## Data Availability

All data generated or analyzed during this study are included in this article.

## Supplementary Materials

The following supporting information can be downloaded at: https://www.mdpi.com/article/10.3390/life13051222/s1, Figure S1: Multiple protein alignment for representatives of four paralogous groups of the *Metridia longa* luciferase isoforms; Figure S2: Multiple sequence alignment of representatives of putative paralogous groups of all known copepod luciferases generated by ClustalW and manually corrected. Table S1: Primers used to obtain MLuc7 deletion mutants by mutagenesis or by cloning of PCR fragments. Figure S3: Denaturing SDS-PAGE analysis (12.5% acrylamide) of expression of MLuc7, some deletion mutants in *E. coli* cells and final high-purity preparations; Coordinates data S1: Predicted structural model for MLuc7wt; Coordinates data S2: Structural model for ML7-N10 deletion mutant of MLuc7. Reference [35] is cited in Supplementary Materials.
