# Supplementary material for: Localization of the Catalytic Domain of Copepod Luciferases: Analysis of Truncated Mutants of the Metridia longa Luciferase
Source: Life (Basel). 2023 May 21;13(5):1222. doi: 10.3390/life13051222 (PMC10220987; doi:10.3390/life13051222)
Supplement: Supplementary file 1 [file life-13-01222-s001.zip › Supplement.pdf]

Electronic Supplementary Information for

Localization of the catalytic domain of copepod luciferases:

Analysis of truncated mutants of the *Metridia longa* luciferase

Svetlana V. Markova[1,2], Marina D. Larionova[1], Igor A. Korotov[1], and Eugene S. Vysotski[1]*

[1]*Photobiology Laboratory, Institute of Biophysics SB RAS, Federal Research Center "Krasnoyarsk Science Center SB RAS", Krasnoyarsk, Russia*

[2]*School of Fundamental Biology and Biotechnology, Siberian Federal University, Krasnoyarsk, Russia*

*Author for correspondence: Eugene S. Vysotski; e-mail <eugene_vysotski@ibp.ru>;
tel. 7 (391) 249-44-30; fax 7 (391) 290-54-90

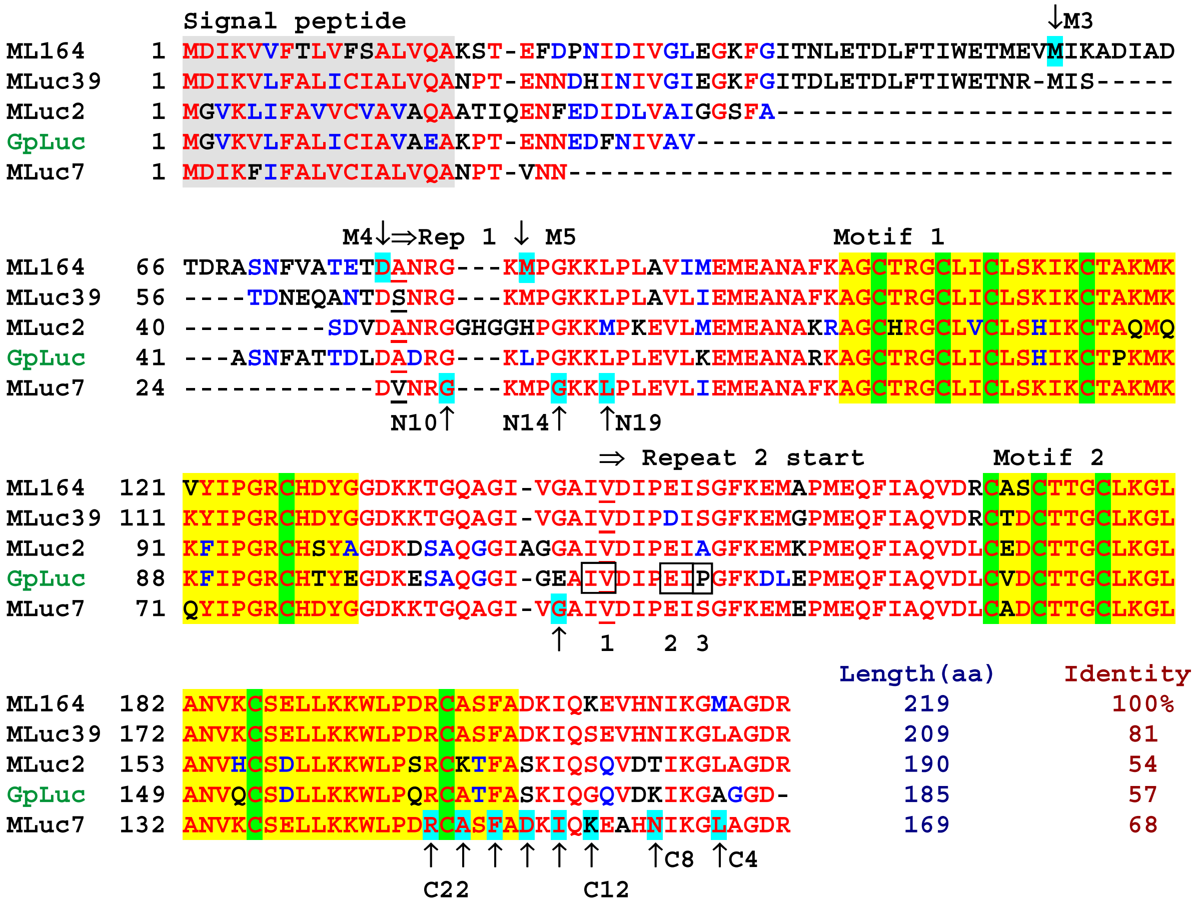

Figure S1. Multiple protein alignment for representatives of four paralogous groups of the *Metridia longa* luciferase isoforms: MLuc164 (AAR17541), MLuc39 (ABW06650), MLuc7 (AJC98141), and MLuc2 (APQ47583), and for the *Gaussia princeps* luciferase GpLuc (AAG54095). The alignment was produced by ClustalW and adjusted manually using nucleotide sequence data. The identical amino acid residues are marked in red; the similar amino acid residues – in blue. The predicted signal peptides providing secretion of the luciferases are shown with a gray box. The start of two non-identical tandem repeats in luciferases' sequences is indicated by horizontal arrow. The most similar motifs within non-identical repeats are marked by yellow, conservative Cys residues are highlighted in green. Bottom vertical arrows and cyan boxes mark the boundaries of the MLuc7 deletion mutants obtained in this work. Start points of the N-terminus truncated mutants of MLuc164 luciferase isoform revealing a high activity [1] are labeled as M3, M4, and M5 with top vertical arrows and cyan boxes. The sites dividing the Gaussia luciferase into specific fragments are framed: 1 – the fragments' edges for protein complementation assay (PCA) [2], 2 – the end-point of GpLuc fragment corresponded to the first repeat with declared bioluminescent activity [3], 3 – the end of the first half of GpLuc with declared high activity [4].

```
                    Predicted signal peptides in 17-22 aa
LoLuc1-3      1 ----MISW--NLFAFATIIALSQALPA------SPTDRSIVLDNGYV-------------CSWEGIP

PsLuc2        1 -----MSI--QFLYALVCLAAAGCQSQKLLPSEDPEQYNIADLDDLVAKLSITDDEMETYTIWE---

PsLuc1        1 -----MYI--KVLFGLTCLSLVLAQPT-----ENKKESYTEDTD----------------------
PaLuc1        1 -----MYI--TVLLGLTCLSLVLAQPT-----ENKQESQIEDIDRSTSLG-------LMCYEQCTGQ
PxLuc1-7      1 -----MYI--KVLLGLTCLSLVLAQPT-----ENKRESDIEDIDRSTSLG-------LMCYQQCTGQ
PxLuc1-8      1 -----MYI--KVWFGLACLSLVLAQPT-----ENKQESHIVDSD---------------------

MLuc164       1 -----MDI--KVVFTLVFSALVQAKST-----EFDPNIDIVGLEGKFGI-TNLETDL--FTIWETME

MLuc39        1 -----MDI--KVLFALICIALVQANPT-----ENNDHINIVGIEGKFGI-TDLETDL--FTIWETNR
MoLuc1        1 MPRGNMDI--KVLFALTCFALVQSNPT-----ETQDGVDILGVEGKFGTETNLETDL--FTIWEING
MpLuc1        1 ----MMEI--KVLFALICFALVQANPT-----ENKDDIDIVGVEGKFGT-TDLETDL--FTIVEDMN
MlLuc-g7      0 -------------------------------------------------------------------

MLuc7         1 -----MDI--KFIFALVCIALVQANPT-----VNN-------------------------------

MaLuc1        1 -----MDI--KVLFALICVAMVQAKAT-----ENNDDIDIVGIASTFI-------------------
McLuc1        1 -----MDI--KVLFALICVALVQAKPT-----ENNDDIDIVGIASTFI-------------------
GpLuc         1 -----MGV--KVLFALICIAVAEAKPT-----ENNEDFNIVAVASNFA-------------------

MaLuc2        1 -----MGV--KLIFAVLCVAVAQAATI----NENFEGIDLVAIGGSFGP------------------
McLuc2        1 -----MGV--KLIFAVLCVAVAQAATI----NENFEGIDLVAIGGSFGP------------------
MLuc2         1 -----MGV--KLIFAVVCVAVAQAATI----QENFEDIDLVAIGGSFA-------------------
MoLuc2        1 -----MGV--KLIFAVLCVAVAQAATI----NENFEDIDLVAIGGSFA-------------------
MlLuc-g1      0 -------------------------------------------------------------------
MpLuc2        1 -----MGV--KLIFAVVCVAAAQAATI----NENFEDIDLVAIGGSFA-------------------
PaLuc2        1 -----MAL--KFLVAVICLAAVQAKSI-----DSYENIDIVAVAGNFAA------------------
PxLuc2        1 -------------------------------------------------------------------

HmLuc1        1 ----MVRLPILVVISLASLYIIQAWAA-----TDEEELDLFDRVK---------------NYWAIGV
HmLuc2        1 ----MFRLPILVVISLASLYIIQAWAA-----TDEEELDLFDRVK---------------NYWGIGV

HtLuc2        1 ----MWRLLSLMLLAVTSVY-IQVWAA-----SEEADDDLVSLVK---------------NYWGVGV
HtLuc1-1-2    1 ----MWHLLSLMLLAVTSVY-MAAL--------EEADDDLVE------------------NYWRIGV
HtLuc1-2-2    1 ----MWHLLSLMLLAVTSVY-IQVVAA-----SEEADDDHVSLVK---------------NYWRIGV
```

```
                                       ↓ Start of repeat 1         Motif 1
LoLuc1-3      43 D-----------DLRDCPKTEDMSKQHG---AALKLPPDVLDEMECNAKKSGCVRGCLQCLALI

PsLuc2        58 -----ELLIISQDFANNLNVVDGDRD--RKLPGKKLPLEVLKIMEANARRAGCTRGCLICLSKI

PsLuc1        33 ------------------VNGDHDRG--RKLPGKKLPLEVLKIMEANARRAGCTRGCLICLSKI
PaLuc1        49 SGLDLKCYKECADF-----TGDRNRG--RKLPGKKLPLEVLKIMEANARRAGCTRGCLICLSKI
PxLuc1-7      49 SGLDLTCYKQCTDV---SGVRDYNRG--KKLPGKKLPLEVLKIMEANARRAGCTRGCLICLSKI
PxLuc1-8      33 --------------------LDGDRG--RKLPGKKLPIEVLKIMEANARRAGCTRGCLICLSKI
                  ↓M3                 ↓M4       ↓M5
MLuc164       53 VMIKADIADTDRASNFVATETDANRG---KMPGKKLPLAVIMEMEANAFKAGCTRGCLICLSKI

MLuc39        52 -MIST---------DNEQANTDSNRG---KMPGKKLPLAVLIEMEANAFKAGCTRGCLICLSKI
MoLuc1        59 -IIKSD-------RDTNRANTDADRG---KMPGKKLPLAVLIEMEANAFKAGCTRGCLICLSKI
MpLuc1        54 -VIS---------RDTNLVNSDADRG---KMPGKKLPLEVLIEMEANARKAGCTRGCLICLSKI
MlLuc-g7      01 ------------------------------------PLEVLIEMEANARKAGCTRGCLICLSKI
                                         ↓N10   ↓N14
MLuc7         24 ---------------------DVNRG---KMPGKKLPLEVLIEMEANAFKAGCTRGCLICLSKI
                                                   ↑N19
MaLuc1        37 -----------------TTNTDADRG---KMPGKRLPLAVLKEMEANAVKAGCSRGCLICLSKI
McLuc1        37 -----------------TTNTDADRG---KMPGKKLPLAVLKEMEANAAKAGCSRGCLICLSKI
GpLuc         37 -----------------TTDLDADRG---KLPGKKLPLEVLKEMEANARKAGCTRGCLICLSHI

MaLuc2        39 ------------------TDVDANRGGHGEMPGQKLPLAVLKEMEANAVRAGCHRGCLICLSHI
McLuc2        39 ------------------TDVDANRGGHGEMPGQKLPLAVLKEMEANAVRAGCHRGCLICLSHI
MLuc2         38 ------------------SDVDANRGGHGGHPGKKMPKEVLMEMEANAKRAGCHRGCLVCLSHI
MoLuc2        38 ------------------TDVDANRGGHGGHPGKKMPKEVLLEMEANAKRAGCHRGCLICLSHI
MlLuc-g1       1 ---------------------------HGGHPGKKMPKEVLLEMEANAKRAGCHRGCLICLSHI
MpLuc2        38 ------------------LDVDANRGGHGGHPGKKMPKEVLVEMEANAKRAGCHRGCLICLSHI
PaLuc2        38 ------------------VDQDANRGG--NLPGKKMPIEVLKEMEANAKRAGCVRGCLICLSHI
PxLuc2         1 ---------------------------------------------------GCTRGCLICLSHV

HmLuc1        44 -------------ANDYDGAVSLDR--KAKLP-KKLSKAVMMEMEANAKEAGCQKSCLICMSKV
HmLuc2        44 -------------ANDYDGTVSLDR--KGKLP-HKLSKAVLIEMEANAQRAGCQRQCLIGLSKI

HtLuc2        43 ---------------SNERDVSLDRGGHGKLP-KKLSVEILAEMEANAQKSNCSRGCLIGLSKI
HtLuc1-1-2    37 ---------------GNERDVSLDRGGPP-----KLTKELLAEMHAIAVNAGCSRVCLIGLSKI
HtLuc1-2-2    43 ---------------GNERDVSLDRGGPP-----KLSKELLAEMHAIASNAGCSRVCLIGLSKI
```

```
                                                    ↓Start of repeat2        Motif 2
LoLuc1-3       93 KCTAKMRKYIPGRCHSYEGDKDIAQGGIGKELTIDIPEIPGFLDLAPMDQFVAQVDLCVDCSSRCLK

PsLuc2        115 KCTAKMKKFIPGRCHTYEGDKSIGQGGIGAA-IIDIPEIPGFKELEPMEQFIAQVDLCADCTTRCLK

PsLuc1         77 KCTAKMKQYIPGRCHTYEGDKSIGQAGIGGP-IIDIPEIIGFKNMEPMEQFIAQVDLCADCTTGCLK
PaLuc1        106 KCTAKMKQYIPGRCHTYEGDKSIGQGGIGGP-IVDIPEIIGFQNMEPMEQFIAQVDLCADCTTGCLK
PxLuc1-7      108 KCTAKMKRYIPGRCHTYEGDKSIGQGGIGGP-IVDIPEIIGFKNMEPMDQFIAQVDLCADCTTGCLK
PxLuc1-8       75 KCTAKMKRYIPGRCHTYEGDKSIGQGGIGGP-IVDIPEIIGFKNMEPMDQFIAQVDLCADCTTGCLK

MLuc164       114 KCTAKMKVYIPGRCHDYGGDKKTGQAGIVGA-IVDIPEISGFKEMAPMEQFIAQVDRCASCTTGCLK

MLuc39        104 KCTAKMKKYIPGRCHDYGGDKKTGQAGIVGA-IVDIPDISGFKEMGPMEQFIAQVDRCTDCTTGCLK
MoLuc1        112 KCTAKMKEYIPGRCHDYGGDKKTGQAGIVGA-IVDIPEISGFKELGPMEQFIAQVDLCADCTTGCLK
MpLuc1        105 KCTAKMKVYIPGRCHDYGGDKKTGQAGIVGA-IVDIPEISGFKELEPMEQFIAQVDLCADCTTGCLK
MlLuc-g7       29 KCTAKMKVYIPGRCHDYGGDKKTGQAGIVGA-IVDIPEISGFKELGPMEQFIAQVDLCADCTTGCLK

MLuc7          64 KCTAKMKQYIPGRCHDYGGDKKTGQAGIVGA-IVDIPEISGFKEMEPMEQFIAQVDLCADCTTGCLK

MaLuc1         81 KCTAKMKQYIPGRCHDYGGDKKTGQAAIEGA-IDDIPEISGFKEMAPMEQFIAQVDLCADCTTGCLK
McLuc1         81 KCTAKMKQFIPGRCHDYGGDKKTGQAALVGA-IFDIPEIFGFLDMEPIEQFIAQVDLCAGCTTGCLK
GpLuc          81 KCTPKMKKFIPGRCHTYEGDKESAQGGIGEA-IVDIPEIPGFKDLEPMEQFIAQVDLCVDCTTGCLK

MaLuc2         85 KCTAKMKQFIPGRCHSYAGDKGSAQGGIDDAAIVDMPEIAGFKDLAPMDQFIAQVDLCEDCTTGCLK
McLuc2         85 KCTAKMKKFIPGRCHSYAGDKGSAQGGIDDSATVDMPEIAGFKDLAPMDQFIAQVDLCKDCTTGCLK
MLuc2          84 KCTAQMQKFIPGRCHSYAGDKDSAQGGIAGGAIVDIPEIAGFKEMKPMEQFIAQVDLCEDCTTGCLK
MoLuc2         84 KCTQKMKKFIPGRCHSYAGDKDSAQGGITEEETVDMPEIAGFKDLEPMEQFIAQVDLCVDCTTGCLK
MlLuc-g1       38 KCTKKMKKFIPGRCHSYAGDKDSAQGGITEEETVDMPEIPGFKDKEPMDQFIAQVDLCVDCTTGCLK
MpLuc2         84 KCTKKMKKFIPGRCHSYEGDKDSAQGGIGEE-IVDMPEIPGFKDKEPMDQFIAQVDLCVDCTTGCLK

PaLuc2         82 KCTAKMKKFIPGRCHSYHGDADTKQGALEE--VVDMPEIPGFVDMEPMEQFIAQVDKCEDCTTGCLK
PxLuc2         14 KCTAKMKKFIPGRCHSYEGDGDTAQGGIEL--VVDMPDIPEFQEMEPMEQFIAQVDKCEDCTTGCLK

HmLuc1         92 KCTKKMKKWLPGRCHAFV--PATDVIPLEPAS-----DIPGYANMTAMQQFNGQVNEC-PCSTRCLK
HmLuc2         92 KCTLKMKKWLPGRCHSYAGDPATGQGPLEPAS-----DIPGYENMTGMQQFNAQVNEC-PCSTRCLK

HtLuc2         91 KCTPKMKKFLPGRCHEYSGDPKTGQGPLTAAA-----VIPGYSDLTAMEQFKLQVDKC-DCSTQCLK
HtLuc1-1-2     81 KCTPKMMTFLPGRCKTFSPNPATGGGPFAAAA-----AIPGFSDLTAMEQYKAQVAQC-DCSSKCLV
HtLuc1-2-2     87 KCTPKMKTFLPGRCNTFAPKPATGDGPFAAAA-----AIPGFSDLTAMEQYKAQVAQC-DCSNRCLV
```

```
LoLuc1-3     160 GLANVQCSCKLYKWLPTRCTGFQAKIKKE-ADTVIGLEDALALGFDTIQACVAAGKCKDTVGRYS

PsLuc2       181 GLANVRCNDLLKKWLPDRCAGFANKIQSE-VHNIKGLAGDR------------------------

PsLuc1       143 GLANVRCNDLLKKWLPDRCAGFALKIQGE-VENIKGMAGDR------------------------
PaLuc1       172 GLANVRCNDLLKKWLPDRCAGFANKIQSE-VDNIKGLAGDR------------------------
PxLuc1-7     174 GLANVRCNDLLKKWLPDRCAGFADKIQNE-VDSIKGMAGDR------------------------
PxLuc1-8     141 GLANVRCNDLLKKWLPDRCAGFADKIQNE-VDSIKGMAGDR------------------------

MLuc164      180 GLANVKCSELLKKWLPDRCASFADKIQKE-VHNIKGMAGDR------------------------

MLuc39       170 GLANVKCSELLKKWLPDRCASFADKIQSE-VHNIKGLAGDR------------------------
MoLuc1       178 GLANVKCSALLKKWLPDRCASFADKIQSE-VHNIKGLAGDR------------------------
MpLuc1       171 GLANVKCSALLKKWLPDRCASFADKIQSE-VDNIKGLAGDR------------------------
MlLuc-g7      95 GL---------------------------------------------------------------
                                  ↓C22      ↓C12 ↓C8 ↓C4
MLuc7        130 GLANVKCSELLKKWLPDRCASFADKIQKE-AHNIKGLAGDR------------------------
                                   ↑ ↑ ↑ ↑C14
MaLuc1       147 GLANVKCSELLKKWLPKRCTSFATKMQKE-IHNIKGMGGDR------------------------
McLuc1       147 GLANIKCSELLKKWLPKRCTSFAYKMQKE-MHNIKGMAGDR------------------------
GpLuc        147 GLANVQCSDLLKKWLPQRCATFASKIQGQ-VDKIKGAGGD-------------------------

MaLuc2       152 GLANVHCSDLLKKWLPSRCKSFATKIQSQ-VDTIKGLAGER------------------------
McLuc2       152 GLANVHCSDLLKKWLPSRCKSFATKIQSQ-VDTIKGLAGAR------------------------
MLuc2        151 GLANVHCSDLLKKWLPSRCKTFASKIQSQ-VDTIKGLAGDR------------------------
MoLuc2       151 GLANVHCSDLLKKWLPSRCKTFASKIQSQ-VDTIKGLAGDR------------------------
MlLuc-g1     105 GLANVHCSDLLKKWLPSRCKTFASKIQSQ-VDTIKGLAGDR------------------------
MpLuc2       150 GLANVHCSDLLKKWLPSRCKTFASKIQSQ-VDTIKGLAGDR------------------------

PaLuc2       147 GLANVHCSDLLKKWLPQRCSQFADKIQSE-VDTIKGLAGDR------------------------
PxLuc2        79 GLANIHCNDLLKKWLPQRCSQFADKIQSE-VDTIKGLGGDR------------------------

HmLuc1       151 GLANIKCSKQLFDAMPGRCKSFRDQILKE-VHKIKGLNDITSAKEAQKQL—DKGK---------
HmLuc2       153 GLANVKCSKQLFDALPTRCRNFRVQIQKE-VHKIKGLNDITSAKEAIKQT—DKGK---------

HtLuc2       152 GLANVKCSAALKAVLPTRCSQFATQIQAE-VGTIKGKGKKPTPPIG-------------------
HtLuc1-1-2   142 GLANIKCSAALKKALPARCTTFKTNIQKEGAVDSIKGYGRK------------------------
HtLuc1-2-2   148 GLANIKCSAALKAALPQRCTTFATNIQKEGEVDSIKGYGRK------------------------
```

Figure S2. Multiple sequence alignment of representatives of putative paralogous groups of all known copepod luciferases generated by ClustalW and manually corrected. Maximally different sequences were used for alignment. In case of several isoforms differing by 1-3 residues, only one consensus sequence was chosen for alignment. E.g., the isoform MLuc2 of *Metridia longa* was determined as a consensus sequence for the group of 8 isoforms [5]. For color designations in the alignment refer to Fig. S1. The end points for the truncated mutants MLuc7 are marked with blue boxes. Start points for the truncated MLuc164 mutants revealing high activity [1] are labeled as M3, M4, and M5. The copepod luciferases in alignment are indicated as: LoLuc1-3 (GenBank accession number BAN91831) from *Lucicutia ovaliformis*; PsLuc1 (BAN91827) and PsLuc2 (BAN91828) from *Pleuromamma scutullata*; PaLuc1 (BAL63034) and PaLuc2 (BAL63035) from *Pleuromamma*

abdominalis; PxLuc1-7 (BAN91829), PxLuc1-8 (BAN91832), and PxLuc2 (BAN91830) from *Pleuromamma xiphias*; MLuc164 (ML164 or MLuc, AAR17541), MLuc39 (ABW06650), MLuc7 (AJC98141), and MLuc2 (APQ47583) from *Metridia longa*; MoLuc1 (BAL63032) and MoLuc2 (AB519699) from *Metridia okhotensis*; MpLuc1 (BAG48249) and MpLuc2 (BAG48250) from *Metridia pacifica*; MaLuc1 (BAN91823) and MaLuc2 (BAN91824) from *Metridia asymmetrica*; McLuc1 (BAN91825) and McLuc2 (BAN91826) from *Metridia curticauda*; MlLuc1-g7 (TRINITY_DN40692_c0_g7_i1) and MlLuc2-g7 (TRINITY_DN49928_c1_g1_i16) from *Metridia luciens* [6]; GpLuc (AAG54095) from *Gaussia princeps*; HtLuc2 (BAL63040), HtLuc1-1-2 (BAL63037) and HtLuc1-2-2 (or HtLuc, BAL63039) from *Heterorhabdus tanneri*; HmLuc1 (BAL63041) and HmLuc2 (BAL63042) from *Heterostylites major*.

Table S1. Primers used to obtain MLuc7 deletion mutants by mutagenesis or by cloning of PCR fragments. Cloning sites in sequences are underlined: NdeI-XhoI for pET22b+ and KpnI-XhoI for pcDNA3m [**Error! Bookmark not defined.**] vectors.

No.	Construct	Primers for MLuc7 deletion mutants
	On the base of pET22b+-MLuc7 or -GpLuc for *E. coli* expression	
1	ML7-C4	Forward T7pro primer and reverse 5'-GATCTCGAGTCATTAAAGACCCTTGATGTTGTGC-3'
2	ML7-C8	Forward T7pro and reverse 5'-GAGCTCGAGTCATTAGTTGTGCGCTTCTTTTGA-3'
3	ML7-C12	Forward T7pro and reverse 5'-ATGCTCGAGTCATTATTTTGAATTTTGTCAGCAA-3'
4	ML7-C14	5'-AGTTTTGCTGACAAAATTTAATGACTCGAGCACCACCA-3' and complementary reverse primer for mutagenesis
5	ML7-C16	Forward 5'-GTGCAAGTTTTGCTGACTAATGACTCGAGCACCAC-3' and complementary reverse primer for mutagenesis
6	ML7-C18	5'-GACAGATGTGCAAGTTTTTAATGACTCGAGCACCACCA-3' and complementary reverse primer for mutagenesis
7	ML7-C20	Forward T7pro and reverse 5'-AGTCTCGAGTCATTATGCACATCTGTCTGGCAG-3'
8	ML7-C22	Forward T7pro and reverse 5'-AGTCTCGAGTCATTATCTGTCTGGCAGCCATTTC-3'
9	ML7-R1	Forward T7pro and reverse 5'-AGTCTCGAGTCATTAACCCACTATTCCAGCCTGT-3'
10	ML7-R2	Forward 5'-CAGTTCATATGGGTGCTATTGTTGACATTCC-3' and reverse #L7: 5'-GATCTCGAGTCATTAACGATCTCCAGCAAGAC-3'
11	ML7-N10	Forward 5'-CAGTGCATATGGGTAAAATGCCTGGGAAAA-3 and #L7 as reverse.
12	ML7-N14	Forward 5'-CAGTGCATATGGGGAAGAAATTGCCACTGGA-3' and #L7 as reverse.
13	ML7-N19	Forward 5'-CTCATCATATGCTGGAAGTACTTATAGAAATGG-3' – and #L7 as reverse.
14	GpLuc	Forward 5'-GACAACATATGAAACCAACTGAAAACAATG-3' and reverse 5'-TTACTCGAGGTTAATCACCACCGGCACC-3'
	On the base of pcDNA3m-MLuc7 [7] for mammalian expression	
14	pcDNA3m-ML7-N10	Forward 5'-TGCATTGGTCCAAGCCGGTAAAATGCCTGGGA-3' and complementary reverse primer, template pcDNA3m-MLuc7
15	pcDNA3m-ML7-N10C4	Template pcDNA3m-ML7-N10 [2], forward T7pro and reverse 5'-GATCTCGAGTCATTAAAGACCCTTGATGTTGTGC-3' primers

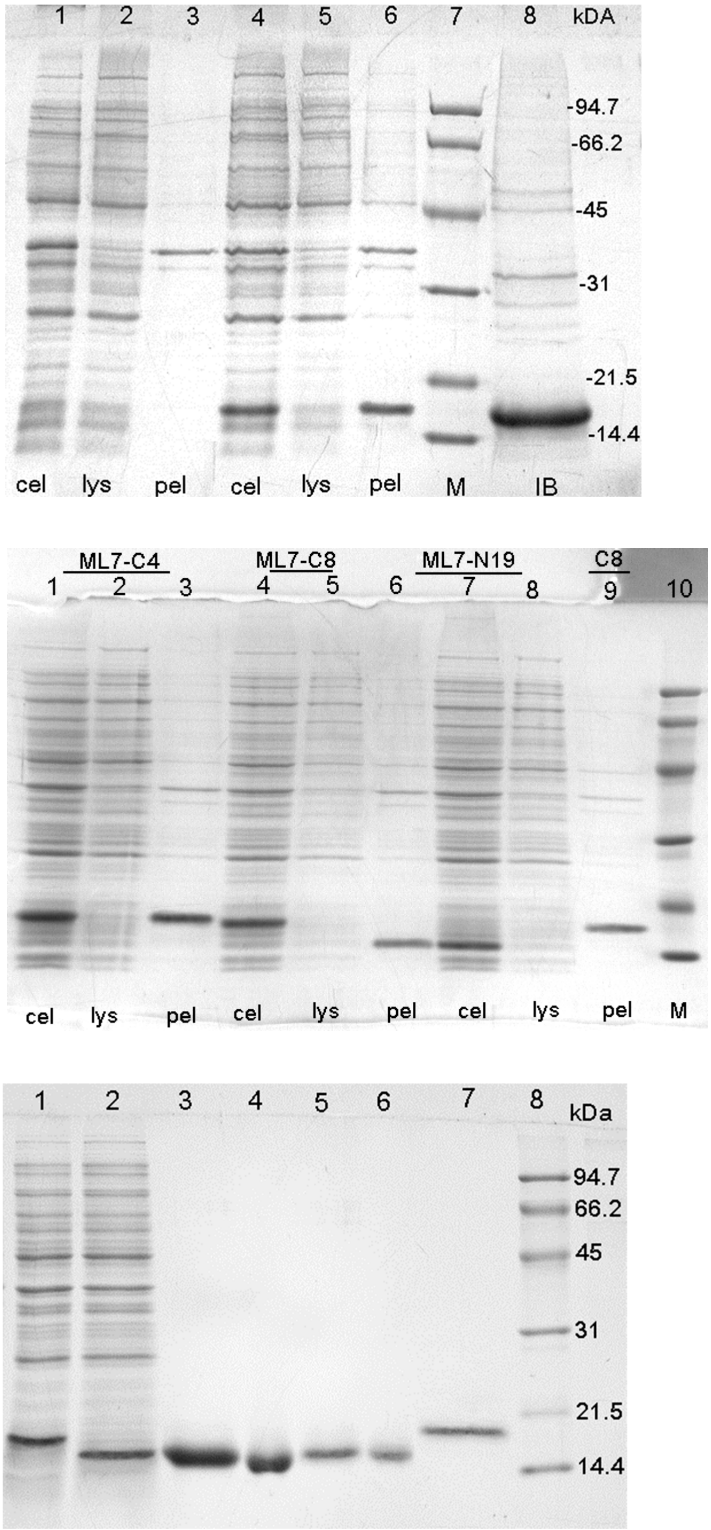

(a)

(b)

(c)

Figure S3. Denaturing SDS-PAGE analysis (12.5% acrylamide) of expression of MLuc7, some deletion mutants in *E. coli* cells and final high-purity preparations: (**a**) Samples of cell fractions. Lanes 1-3, control *E. coli* cells: 1, whole cells; 2, soluble fraction; 3, insoluble cell pellet. Lanes 4-8, MLuc7 expression: 4, whole cells; 5, soluble fraction; 6, insoluble cell pellet; 7, low range protein standards (Bio-Rad); 8, washed insoluble cell pellet (inclusion bodies) for preparative MLuc7

purification; (**b**) Samples of MLuc7 deletion mutant expression. Lanes 1-3, ML7-C4 expression: 1, whole cells; 2, soluble fraction; 3, insoluble cell pellet. Lanes 4-5, ML7-C8 expression; 4, whole cells; 5, soluble fraction. Lanes 6-8, ML7-N19 expression: 6, insoluble cell pellet; 7, whole cells; 8, soluble fraction. Lane 9: insoluble cell pellet with ML7-C8 synthesis. Lane 10: low range protein standards (Bio-Rad). Polyacrylamide gels were stained with Coomassie blue; (**c**) Lanes: 1, *E. coli* expressing MLuc7; 2, *E. coli* expressing ML7-N10; final luciferase preparations: 3, ML7-N10; 4, semi-native PAGE of non-denatured ML7-N10; 5, ML7-N10C4; 6, semi-native PAGE of non-denatured ML7-N10C4, 7, MLuc7; 8, low range protein standards (Bio-Rad). The sample for semi-native gel electrophoresis was prepared without reducing agents (DTT and β-mercaptoethanol) addition and heating.

Coordinates data S1. Predicted structural model for MLuc7wt. Prediction of the structure was carried out using the on-line server I-TASSER [8–10]. As a reference model, the structure of Gaussia luciferase (PDB ID 7D2O) was used. The file is attached to the supplementary files' archive as MLuc7wt.pdb.

Coordinates data S2. Structural model for ML7-N10 deletion mutant of MLuc7. Prediction of the structure was carried out using the on-line server I-TASSER [8–10]. As a reference model, the structure of Gaussia luciferase (PDB ID 7D2O) was used. The file is attached to the supplementary files' archive as ML7N10.pdb.

References

1. Markova, S.V.; Burakova, L.P.; Vysotski, E.S. High-active truncated luciferase of copepod *Metridia longa*. *Biochem Biophys Res Commun* **2012**, *417,* 98-103. https://doi.org/10.1016/j.bbrc.2011.11.063.
2. Remy, I.; Michnick, S.W. A highly sensitive protein-protein interaction assay based on Gaussia luciferase. *Nat Methods,* **2006**, *3,* 977-979. https://doi.org/10.1038/nmeth979.
3 Inouye, S.; Sahara, Y. Identification of two catalytic domains in a luciferase secreted by the copepod Gaussia princeps. *Biochem Biophys Res Commun* **2008**, *365,* 96-101. https://doi: 10.1016/j.bbrc.2007.10.152.
4 Hunt, E.A.; Moutsiopoulou, A.; Ioannou, S.; Ahern, K.; Woodward, K.; Dikici, E.; Daunert, S.; Deo, S.K Truncated variants of Gaussia luciferase with tyrosine linker for site-specific bioconjugate applications. *Sci Rep* **2016**, *8*, 26814. https://doi: 10.1038/srep26814.

5 Larionova, M.D.; Markova, S.V.; Vysotski, E.S. The novel extremely psychrophilic luciferase from *Metridia longa*: Properties of a high-purity protein produced in insect cells. *Biochem Biophys Res Commun* **2017**, *483,* 772-778.

6 Tessler, M.; Gaffney, J.P.; Crawford, J.M.; Trautman, E.; Gujarati, N.A.; Alatalo, P.; Pieribone, V.A.; Gruber, D.F. Luciferin production and luciferase transcription in the bioluminescent copepod *Metridia lucens*, *Peer J* **2018**, *6*, e5506. https://doi: 10.7717/peerj.5506.

7 Markova, S.V.; Larionova, M.D.; Burakova, L.P.; Vysotski, E.S. The smallest natural high-active luciferase: cloning and characterization of novel 16.5-kDa luciferase from copepod Metridia longa. *Biochem Biophys Res Commun* **2015**, *457*, 77-82. https://doi:10.1016/j.bbrc.2014.12.082

8 Zhang, Y. I-TASSER server for protein 3D structure prediction. *BMC Bioinform* **2008**, *9*, 40. https://doi.org/10.1186/1471-2105-9-40

9 Roy, A.; Kucukural, A.; Zhang, Y. I-TASSER: A unified platform for automated protein structure and function prediction. *Nat Protoc* **2010**, *5*, 725–738. https://doi.org/10.1038/nprot.2010.5

10 Yang, J.; Yan, R.; Roy, A.; Xu, D.; Poisson, J.; Zhang, Y. The I-TASSER Suite: Protein structure and function prediction. *Nat Methods* **2015**, *12,* 7–8. https://doi.org/10.1038/nmeth.3213
